# Patient perspectives on premature termination of eating disorder treatment: a systematic review and qualitative synthesis

**DOI:** 10.1186/s40337-022-00568-z

**Published:** 2022-03-16

**Authors:** Cecilia Vinchenzo, Vanessa Lawrence, Catherine McCombie

**Affiliations:** Department of Health Services and Population Research, Institute of Psychiatry, Psychology and Neuroscience, King’s College London, The David Goldberg Centre, De Crespigny Lane, PO26, London, SE5 8AF UK

**Keywords:** Eating disorders, Qualitative, Dropout, Attrition, Treatment, Patient perspectives

## Abstract

**Background:**

High rates of premature treatment termination are a well-reported issue in eating disorder treatment, and present a significant barrier for treatment effectiveness and longer term health outcomes of patients with eating disorders. Understanding patient perspectives on this phenomenon is essential in improving treatment completion rates and informing research and intervention development. The aim of this review is to synthesise qualitative literature on patient perspectives of premature termination of eating disorder treatment and to summarise the key issues leading to discontinuation of treatment.

**Methods:**

A systematic review of 1222 articles was conducted to identify studies using qualitative methods to investigate patient experiences of prematurely terminating eating disorder treatment. Ten articles were included in the review, with thematic synthesis used to analyse the primary research and develop overarching analytical themes.

**Results:**

Conflict around enmeshment of eating disorder with identity, and lack of support with reconstructing a sense of self without the eating disorder; challenges of managing pressures of social and clinical relationships while feeling unheard and misunderstood by both; expectations and disappointments around treatment; and dissatisfaction with progress were key themes behind premature termination of treatment.

**Conclusions:**

The findings of this review demonstrate the key issues influencing the decision to end treatment early, highlighting the contribution of individual, environmental, and service-level factors. Implications of these factors are discussed and suggestions raised for future research and service development.

## Plain English Summary

Understanding why people decide to leave treatment for an eating disorder is an important part of developing effective eating disorder treatment. Treatment must fully take into account patients’ needs and be acceptable to them. This review summarises research into patient perspectives on dropping out of eating disorder treatment, with the aim of identifying issues leading to dropout that have been raised across multiple studies. We found ten articles which asked patients to talk about their treatment experience and why they decided to leave treatment before it was complete. There were several key concerns reported by patients. Firstly, patients reported feeling that an eating disorder had become part of their identity, and that treatment tried to remove that without helping them build an identity without the eating disorder. Secondly, difficulties around feeling unheard and misunderstood in both social and clinical relationships were reported, that left patients feeling unsupported in treatment. Finally, patients expressed overall disappointment with the treatments offered and dissatisfaction with progress made. This study provides important understanding of the issues around treatment dropout that will be valuable when planning research, future treatment, and service provision.

## Introduction

Eating disorders are severe and disabling conditions associated with significant psychiatric co-morbidities and high relapse and mortality rates [1, 2]. Providing effective treatment is important for improving health outcomes for these patients. Despite the development of various efficacious treatments, dropout rates within this patient group are the highest amongst all psychiatric conditions [3–5]. Reviews have shown that around 40% of people receiving treatment for an eating disorder do not finish the full course of treatment, with this figure rising to 50% for people with anorexia receiving inpatient treatment [6, 7]. A meta-analysis of cognitive behavioural therapy (CBT) in all eating disorders reported an estimated dropout rate from CBT of 24% [8]. Premature termination of treatment (PTT), also know as ‘dropout’ and ‘attrition’, references a patient’s independent decision to terminate treatment or early discharge by staff [6, 8, 9]. However, variation in definitions is reported to impede PTT rate estimations and make cross-study comparisons difficult [8, 10].

High PTT rates present serious barriers for treatment effectiveness, foster poor health outcomes, and present a considerable burden on patients, their families and health services [6]. Individuals who terminate treatment early have increased likelihood of enduring a more chronic and severe condition [11, 12], experiencing poorer social functioning overall [13] and greater risk of relapsing [14]. The individual is also less likely to seek further treatment [15]. These individual-level impacts also culminate in considerable economic implications for health services [16]. Furthermore, research affected by large PTT rates may lack validity, reliability and generalisability due to attrition bias if certain groups are not completing treatment [15], which has implications for study designs and treatment development [6, 17]. Most critically, patients are not receiving the care they need. While there is much debate and uncertainty about effectiveness of eating disorder treatment, engagement in treatment has been found to aid recovery [18]. Supporting patients to remain in treatment, and the development of treatments that patients find more acceptable, is therefore imperative in improving outcomes for this population.

Research has identified several factors that may influence PTT including: age, treatment modality, illness chronicity, eating disorder sub-type, psychiatric co-morbidity, poor therapist alliance, and service-related factors (e.g. long waiting times) [7, 9, 10, 19–22]. Although these studies were able to detect significant factors influencing PTT, all concluded that small sample sizes, few reliable study replications and lack of heterogeneity across patient populations make it difficult to draw firm conclusions on the nature of and motivations for treatment dropout [7–10].

The value of exploring patients’ views toward treatment and the use of in-depth qualitative methodologies to understand the motives driving PTT was raised by Mahon as being central to understanding PTT beyond risk factors and patient characteristics [15]. Qualitative research may reveal underlying motivations and negative treatment experiences not captured by quantitative studies, such as in Rance et al.’s interview study that revealed people with anorexia nervosa are largely unsatisfied with treatment and the focus on food and weight restoration [23]. Since Mahon’s recommendation, there have been several qualitative studies looking to understand premature termination of eating disorder treatment from patients’ perspectives.

The aim of this review is therefore to synthesise qualitative literature on patient experiences of premature termination of eating disorder treatment, to summarise the central elements driving this phenomenon, and inform interventions to maximise treatment engagement [24, 25]. To the authors’ knowledge, this is the first qualitative systematic review of this topic.

## Methods

This systematic review was conducted in February 2020 following the Preferred Reporting Items for Systematic Reviews and Meta-Analyses (PRISMA) guidelines [26]. The protocol is registered on Prospero, registration number CRD42020172492.

### Data sources and search strategy

A systematic literature search was conducted using electronic databases: MEDLINE, PsycINFO, EMBASE and Cumulative Index to Nursing and Allied Health Literature (CINAHL), and backward citation searching. The search strategies and basis for eligibility criteria were formed using the ‘Sample, Phenomenon of Interest, Design, Evaluation and Research type’ tool [27]. The search strategy employed in this review covered three central concepts: (1) eating disorders, (2) dropout, and (3) qualitative research methodology. Search terms were tailored per database and a mixture of subject heading, key word and free-text searches were piloted to maximise search sensitivity whilst preserving adequate specificity.

### Eligibility criteria

For inclusion in this review, studies needed to meet the following criteria: (a) examined patient-reported experiences of dropout from any form of eating disorder treatment; (b) qualitative data collection or analysis, or mixed methods if the qualitative aspect was separately reported; (c) participants of any age or sex with an eating disorder diagnosis meeting DSM or ICD criteria; (d) dropout definition was provided. For the purpose of this review dropout was defined as a patient’s choice to terminate treatment early or a clinician-led decision to discharge before treatment was complete, after attending at least one session. Definitions of dropout vary widely, and thus comparable definitions were eligible. Data from anyone other than patients are not reflected in this review, such as carers, family or staff. No restrictions on publication date were applied, review articles were eligible. Eligible studies needed to be published in a peer-reviewed journal and written in English. Studies with either patient or clinician-initiated dropout were included, as the aim was to capture the patient experience of dropout regardless of circumstance.

### Study selection and data collection

Titles, abstracts and full texts were screened by CV. A fourth reviewer, external to the review, independently double-screened 10% of the articles. CM finalised eligible studies and facilitated the resolution of reviewer discrepancies. Studies were excluded at the title and abstract stage if there was no mention of investigating treatment dropout in the abstract. Reasons for exclusion at full text stage included incorrect population, no qualitative element, not relevant to dropout, not English language, and full text not accessible. Data extracted from full text reviewing included dropout, treatment intervention, study design, methods, sample features, participant demographic data, key findings, themes, author interpretations and explanations, participant quotes, and theories underpinning the research.

### Quality assessment

CV and CM determined the methodological quality and risk of bias of the included studies using the Critical Appraisal Skills Programme (CASP; 2018) qualitative research evaluation tool. Studies were categorised as low (0–4), medium (5–7) or high (8–10) quality. The usefulness of qualitative research quality appraisal in systematic reviews is debated [28, 29], and thus, no studies were excluded on the basis of the quality assessment. Quality assessment was used to recognise areas of methodological weakness to better interpret findings and inform the overall strength of this review’s body of evidence.

### Data synthesis

Thematic synthesis was used to analyse the primary qualitative research, chosen as it enables ease of qualitative data summarisation, and is useful for comparing experiences, facilitating a new analysis whilst offering a comprehensive interpretation [30]. Data was free coded inductively according to the meaning, context and themes of the primary studies. Similarities and differences between codes were considered and comparable codes grouped into descriptive sub-themes that depicted an aspect of the PTT experience. For example, ‘lack of control’ and ‘feeling powerless’ in treatment were regarded as associated concepts pertaining to the same descriptive sub-theme of ‘control’. Efforts were made to preserve original concepts and terminology, and to refrain from over-combining codes in order to avoid misinterpreting their original contexts. The descriptive sub-themes were compared and the relationship between them explored in relation to the research objectives. This led to the development of overarching analytical themes that were felt to offer a new synthesis of the data.

## Results

### Study selection

**Fig. 1:**
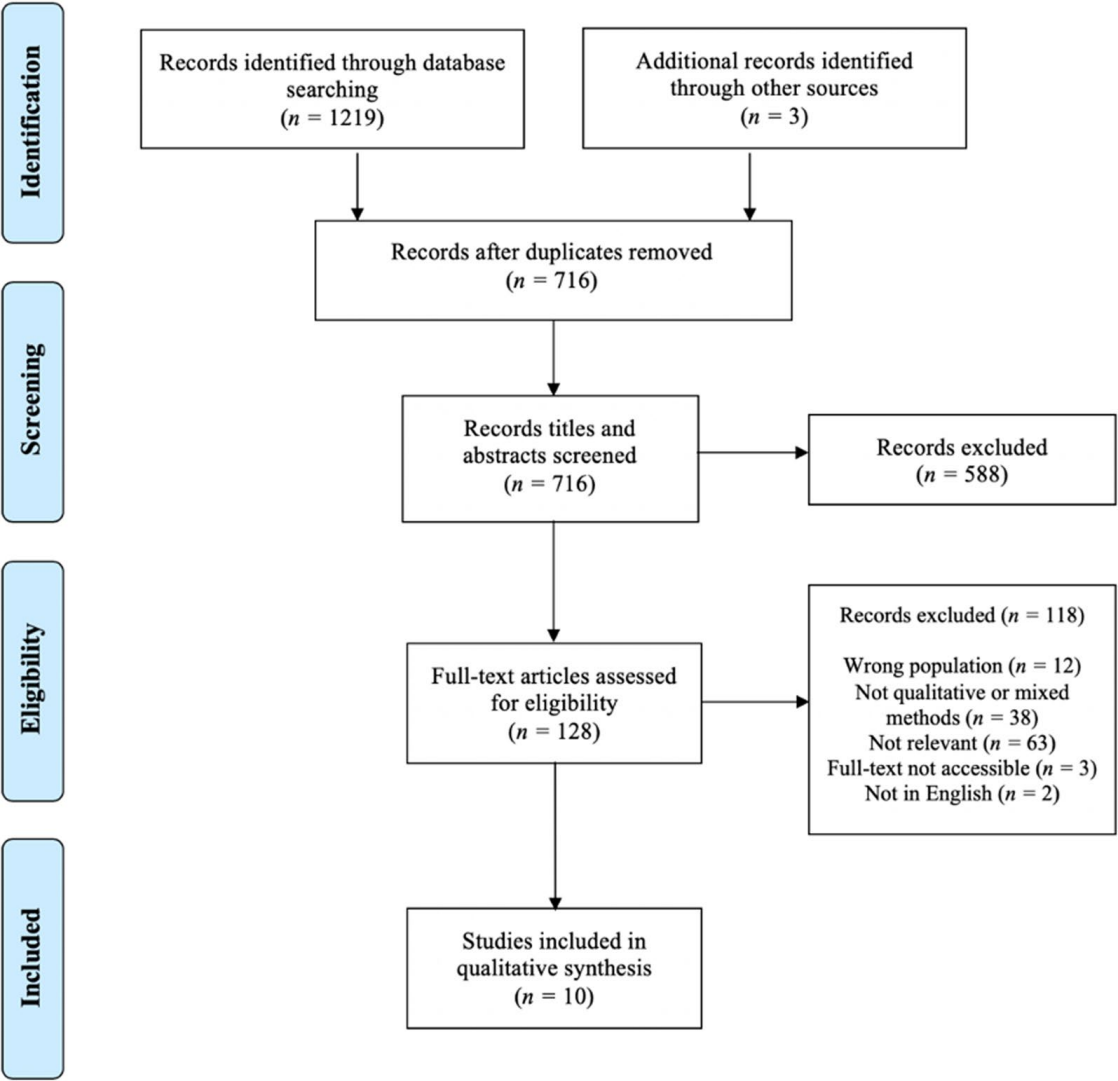
PRISMA flow diagram summarising the systematic review literature identification, screening process, exclusion details and study selection

In total, 1222 potential studies were yielded through database literature searches (*n* = 1219) and online resource ResearchGate (*n* = 3) (Fig. 1). 716 studies remained after de-duplication. After screening titles and abstracts, 128 articles underwent full text review. Ten articles met the inclusion criteria and were incorporated in the review. A PRISMA flow diagram of the identification, screening, and selection process is presented in Fig. 1.

### Study characteristics

The ten studies comprised a total sample of 225 patients, a mixture of inpatient and outpatient treatment settings, eating disorder diagnoses and age ranges, and predominantly women. The studies were primarily from Europe (70%), North America (20%), and South America (10%). Participant sample sizes ranged from 5 to 58. Table 1 provides a summary of the included studies.

**Table 1:** Summary of studies and study characteristics included in the review (in chronological order)

Study	Country	Aims	Definition of dropout	Sample	Method	Treatment setting	Quality assessment rating
Merrill et al. [40]	USA	Compare the characteristics of those who dropout and those who do not in patients with BN receiving group therapy	Dropping out before 20 weeks of completed group therapy	*n* = 17 Gender: N/R Age range: 18–48 Diagnoses: BN Dropout from: Group therapy Ethnicity: Asian = 1, Caucasian = 16 Education level: N/R	Questionnaire assessing dropout reasons with an open-ended question. No further clarification of analysis methods offered	Outpatient	Medium
Eivors et al. [32]	United Kingdom	Understand the meaning of dropout from services for patients with AN	Unilateral decision to dropout made by the patient	*n* = 8 Gender: Women Age range: 21–43 Diagnoses: AN, Partial syndrome AN Dropout from: Specialist adult ED service—outpatient or inpatient Ethnicity: N/R Education level: N/R	Semi-structured interview, written autobiographical account Grounded theory	Participants were treated by a multi-disciplinary outpatient treatment team, with two also treated as inpatients	High
Darcy et al. [36]	USA	Explore how individuals with AN engage in treatment and describe recovery	Non-compliance or treatment failure	*n* = 20 Gender: Women Age range: 19–52 Diagnoses: AN Dropout from: Any ED treatment Ethnicity: Biracial = 3, Caucasian = 17 Education level: N/R	Semi-structured interview with open-ended and probe questions, focus groups, self-report questionnaire Thematic analysis	Treatment was reported by participants as a mix of outpatient and inpatient treatment	High
Vandereycken and Devidt [39]	Belgium	To gain a greater understanding of the meaning dropout from ED therapy from staff and patients	Cessation of treatment in contradiction of the treatment agreement or a unilateral decision to dropout made by the patient	*n* = 19 Gender: Female Age range: 15–35 Diagnoses: AN-R (*n* = 7), AN-BP (*n* = 3), BN (*n* = 6), EDNOS (*n* = 5) Dropout from: Specialist ED inpatient setting Ethnicity: N/R Education level: N/R	Self-report questionnaire, written autobiographical statements from patients Descriptive statistics and written presentation of the qualitative results	Inpatient	Low
Leavey et al. [33]	United Kingdom	Understand the reasons behind non-engagement at a specialist ED unit	Failure to attend first appointment or attended once then dropped out	*n* = 13 Gender: Women (*n* = 12), men (*n* = 1) Age range: N/R Diagnoses: AN, BN, BED Dropout from: GP referral for specialist ED service Ethnicity: Black Caribbean = 1, Black European = 1, Jewish = 1, Turkish Cypriot = 1, White and Asian = 1, White British = 7, White Irish = 1 Education level: N/R	Interview (type and structure not specified) Interpretative phenomenological analysis	Outpatient	High
Seidinger-Leibovitz et al. [34]	Brazil	Explore the meaning of dropout in an outpatient ED setting using qualitative methods	Unilateral decision to dropout made by the patient after attending at least one month of treatment	*n* = 8 Gender: Women Age range: 18–30 Diagnoses: AN-R, AN-P, BN, EDNOS Dropout from: Outpatient ED service Ethnicity: N/R Education level: Graduate = 3, High School = 2, Vocational School = 1, Incomplete High School = 2	Semi-structured interview Thematic analysis using a psychodynamic theoretical framework	Outpatient	High
ter Huurne et al. [31]	Netherlands	Explore reasons for dropout, predictive factors of dropout and investigate the overall patient experience of the treatment of a web-based ED treatment	Did not begin the programme or terminated their attendance during treatment	*n* = 53 Gender: Women Age range: 38.1 ± 12.4 Diagnoses: BN, BED, EDNOS Dropout from: Web-based CBT Ethnicity: N/R Education level: N/R for interviews	Online self-report questionnaire including open-ended question exploring reasons for dropout Descriptive percentages of the qualitative data	Web-based Cognitive Behavioural Therapy	Low
Frostad et al. [38]	Norway	Measure BMI changes in a group of patients with AN receiving a CBT-E intervention	Not starting or dropping out before completing 12 months of a CBT-E programme	*n* = 22 Gender: Women (*n* = 21), men (*n* = 1) Age range: 21.1 ± 4.2 Diagnoses: AN Dropout from: CBT-E Ethnicity: N/R Education level: N/R	Reasons for dropout assessed in detail with therapist and documented Descriptive presentation of the qualitative data	Online	Medium
del Barrio et al. [37]	Spain	Investigate the rate and personal characteristics associated with dropping out from treatment at a 2-year follow-up in a sample of patients diagnosed with an ED	Nonconsensual interruption of treatment ensuing from the patient’s own decision	*n* = 58 Gender: Mixed, *n* per gender unspecified Mean age: 28.3 (*SD* = 9.67) Diagnoses: AN, BN, EDNOS Dropout from: Specialist ED unit, including outpatient therapy Ethnicity: Cauasian = 54, Hispanic = 3, Romani = 1 Education level: N/R	Longitudinal prospective cohort follow-up study, close-ended questionnaire, semi-structured telephone interview Descriptive percentages of the qualitative data	Inpatient	Medium
Bakland et al. [35]	Norway	Investigate the experiences of those who dropped out from a novel specialised ED treatment	Non-completion of less than 80% of the treatment programme	*n* = 5 Gender: Women Age range: 21–41 Diagnoses: BN (*n* = 3), BED (*n* = 2) Dropout from: Combined group therapy, exercise and diet programme Ethnicity: N/R Education level: N/R	Open-ended interview Hermeneutical phenomenology, interpretation theory	Outpatient	High

Key: N/R, Not reported; ED, Eating disorder; AN, Anorexia nervosa; AN-R, Anorexia nervosa restrictive subtype; AN-BP, Anorexia nervosa binge-purge subtype; BN, Bulimia nervosa; BED, Binge eating disorder; EDNOS, Eating disorder not otherwise specified; CBT, Cognitive behaviour therapy; CBT-E, Enhanced cognitive behaviour therapy

NB: Total number of patients for each gender and diagnosis detailed only where available. The total number of patients in a sample expressed is the number of participants who had satisfied the definition of dropout for the respective study and had been assessed using the relevant qualitative method. Data from anyone other than patients (e.g. carers, family or staff) are not reflected in this review

### Quality assessment

Five studies were determined to be of high quality and five of medium quality. Lower scoring aspects included the reporting of ethical considerations and consideration of the researcher-participant relationship.

### Descriptive and analytical themes

The overall objective of this systematic review was to synthesise patient self-reported reasons for dropping out from eating disorder services. Fourteen descriptive sub-themes were identified, grouped into four analytical themes: (1) ‘inner conflict’, (2) ‘connection and communication’, (3) ‘treatment service experience’, and (4) ‘treatment progress’ (Table 2).

**Table 2:** Summary of analytical and descriptive themes and their primary study source

Analytical theme	Descriptive sub-themes	Source
Inner conflict	Loss of control	Eivors et al. [32], Leavey et al. [33], Seidinger-Leibovitz et al. [34], ter Huurne et al. [31]
	Psychological difficulties	Leavey et al. [33], ter Huurne et al. [31]
	Fear	Darcy et al. [36], Leavey et al. [33], Bakland et al. [35]
	Threats to self-identity	Eivors et al. [32], Leavey et al. [33], Seidinger-Leibovitz et al. [34]
	Denial and cognitive dissonance	Eivors et al. [32], Leavey et al. [33], Seidinger-Leibovitz et al. [34]
	Low motivation	Merrill et al. [40], Darcy et al. [36], Vandereycken and Devidt [39], ter Huurne et al. [31], Frostad et al. [38], del Barrio et al. [37]
	Stigma	Eivors et al.[32], Leavey et al. [33]
Connection and communication	Family conflict	Vandereycken and Devidt [39], Leavey et al. [33], Seidinger-Leibovitz et al. [34], ter Huurne et al. [31]
	Tensions in patient-clinician relationship	Eivors et al. [32], Darcy et al. [36], Leavey et al. [33], ter Huurne et al. [31]
	Feeling misunderstood or unheard	Merrill et al. [40], Eivors et al. [32], Leavey et al. [33], Bakland et al. [35]
Treatment service experience	Unmet expectations	Eivors et al. [32], Darcy et al. [36], Vandereycken and Devidt [39], Leavey et al. [33], Seidinger-Leibovitz et al. [34], ter Huurne et al. [31], del Barrio et al. [37], Bakland et al. [35]
	Feeling unsafe in treatment	Eivors et al. [32], Darcy et al. [36], Vandereycken and Devidt [39], Bakland et al. [35]
Treatment progress	Satisfactory progress	Vandereycken and Devidt [39], ter Huurne et al. [31], Frostad et al. [38], del Barrio et al. [37]
	Obstacles to progress and accessibility	Merrill et al. [40], ter Huurne et al. [31], Frostad et al. [38], del Barrio et al. [37]

### Theme 1: Inner conflict

Many patients with eating disorders experienced profound psychological turmoil generated by complex psychosocial and personal difficulties, influencing their decision to leave treatment.

#### Loss of control

Several studies identified the feeling of needing control as a major factor contributing to PTT – both apprehension around experiencing an absence of control and the inherent need to regain it [31]; one patient claimed: “*It was the only thing I felt I could control and still wasn’t ready to give it up*” [32]. Patients felt receiving treatment implied handing over control to clinicians, resulting in distress around ‘giving up’ their disorder, revealing the self-conflict experienced by some patients when deliberating whether to engage in potentially life changing treatment or to continue current behaviours [33, 34]. One patient described stepping back from services in order to restore a sense of self-sovereignty and stated, “*I don’t want them to be able to know me inside out anymore*” [32]. Leaving treatment seemed to provide a sense of relief in these instances [34]. Patients described feeling like having an eating disorder was similar to the powerful, controlling hold of a substance addiction—the struggle of overcoming this ‘addiction’ led to the decision to terminate treatment [34].

#### Co-morbid psychological difficulties

Struggles with co-morbid psychological difficulties led to many patients terminating their treatment early, with eating disorders typically referenced as coping strategies to manage other psychological difficulties. Depressive episodes meant patients did not wish to continue treatment [31]. Long, debilitating periods of poor mental health left patients struggling to leave home and attend services, with travelling to appointments inducing anxiety and fear of leaving the ‘safe space’ of home: “*I found it incredibly difficult to go out.* […] *so I was missing my appointments*” [33]. Some identified their eating disorder as a stress-reliever that allowed them to avoid other challenging emotions such as low mood, stress or low self-esteem [33]. Letting go of this coping mechanism may be felt as impossible, leading to treatment termination.

#### Fear

An intense fear of weight gain led some patients to terminate treatment prematurely [35]: “*Just the feeling of* [*being*] *forced to gain weight. It was kind of just scary for me*” [36]. Some patients left treatment as they feared rejection from others, with some linking this to traumatic experiences and abandonment in childhood: “*I’m scared that if I let too many people come in my life they will go away again*” [33]. Patients evaded treatments which induced these emotions.

#### Threats to self-identity

Several studies demonstrated a profound assimilation of eating disorders with self-identity: “*I see it as a lifestyle that I*’*m going to die with*” [34]. Even after partial recovery, the enduring identity entanglement led to a persistent reminder that they may return to familiar habits: “*It is part of me.* […] *I could just if I wanted to just turn to it and go right back down*” [32]. The eating disorder gave a sense of purpose and life without it was deemed inconceivable, and with the notion of treatment threatening their self-identity, leaving treatment appeared to be an act of self-preservation [33].

#### Denial and cognitive dissonance

Some patients denied illness or experienced cognitive dissonance towards their condition. Despite serious health warnings patients denied dysfunction, reduced eating disorders to simply lifestyle preference, believed their issues to be only physical, or felt they did not fit the image of an eating disorder patient, and thus did not require treatment – all fuelling a sense of doubt and ambivalence towards receiving treatment [32, 33]. One patient stated: “*I watch people who eat normally*, *I think that they are different* [*…*] *and I*’*m normal”* [34]. This sentiment was echoed further: “*I was far too fat to be at a clinic like this, I thought I would be laughed at*” [32]. Patients minimised their conditions, comparing themselves to others whom they judged to be more deserving of care: *“I felt that mine was nothing compared to hers*” [33]. When patients did not believe they had a problem or that others were more deserving, they denied the need for help; this resulted in them leaving treatment.

#### Low motivation

Low levels of motivation for and enthusiasm towards treatment were highlighted. Patients reported feeling like they could not be helped or did not believe treatment could help them, alongside a lack of motivation, feelings of lost hope, low self-confidence, the desire to return to a ‘normal life’ and a reluctance to initiate active therapy [31, 37–40]. Patients who had prematurely terminated treatment recounted becoming increasingly tired of their treatment or experiencing homesickness: “*I was really lonely and really wanted to go home…*” [36].

#### Stigma and shame

Stigma related to an eating disorder diagnosis and associated treatment was recognised as a motivation for leaving treatment: “*The fact that it was a Mental Health Hospital and I obviously thought they were saying there was something wrong with me in the head*” [32]. Being seen by mental health professionals, such as psychiatrists, was negatively perceived and was felt to imply one having a severe psychological disorder, further coupled with a misconception that all of their problems would be held on ‘records’ [33]. Feelings of shame were detailed: “*When I first arrived at the hospital I was so ashamed, I was far too fat to be at a clinic like this, I thought I would be laughed at*” [32]. Coping with the internal stigma of an eating disorder diagnosis and being in treatment for this may lower patients’ confidence, leading to premature treatment termination.

### Theme 2: connection and communication

Patients’ decisions to leave treatment were influenced by their relationships with others, including staff, other patients, and family and friends.

#### Managing multiple pressures

Lack of social support and family tensions were found to contribute to PTT [33]. Difficulties with relatives [41], challenging home lives, and pressure from family members were implicated [39]. One patient described feeling persecuted by family: “[*…There*] *was a lot of pressure*, *the main reason I quit* [*…*] *I do not like my mother*’*s demands* [*… it*] *oppressed me a bit*” [34]. Terminating treatment provided respite from perceived continuous surveillance by family and clinicians, which patients reported as feelings extremely intrusive [34].

#### Feeling misunderstood or unheard

Many patients left treatment due to feeling misunderstood or unheard by clinicians; they felt staff were not able to see their perspective. One patient considered clinicians to be well-intentioned but put in little effort to meet needs as they did not completely understand their problems, amplifying her wariness towards services: “*I just didn’t feel that they truly understood what it’s like to be like that, all these quick fixes* […] *it’s not as simple as that*” [33]. Patients expressed leaving group therapy due to feelings of not belonging within the group [40], feeling unheard and unable to share their experiences, as therapy dialogue often became focussed around one patient: “*Three of the others in my group took a lot of space.* […] *often found myself in a listening position. I had a lot to say but I was never able to share my thoughts*” [35]. Some patients left treatments where they felt therapists lacked receptiveness to their individual needs: “*I don’t think he was sensitive enough to see what I really needed. He just kept focussing on the eating* […]” [32].

### Theme 3: treatment service experience

Many patients decided to prematurely terminate treatment due to perceived unmet needs and expectations, negative experiences, and feeling unsafe during treatment.

#### Unmet expectations

Patients commonly expected a greater degree of personalised and clinician-led guidance than was received, and were left disappointed and desiring more detailed strategies [31, 33, 35]. Several dropped out due to dissatisfaction with their therapeutic programme [36], the quality of the service [37], or because they felt treatment was not offering new insights [31]. Others felt treatments were hyper-focussed on food, body image, or weight tracking and ignored other important aspects of their condition, such as emotions [32, 34]. The desire to move away from this approach was evident: “*To talk about other things unrelated to food.* […] *I wanted to talk about why I*’*m sad*” [34]. Concentrating around these factors may unintentionally support coping methods that centre life around food, perpetuating eating disorders and ultimately, non-engagement [32].

Insufficient clinician contact was also cited as problematic for patients [31]: “*Too many days where I had to do everything by myself.* […] *I needed a more intensive treatment*” [35]. Patients called for a greater utilisation of individual therapy [39] as group therapy was deemed unsuitable due to the wide-ranging group demographics, coupled with varying degrees of illness severity, and concern that others’ inexperience in therapy negatively impacted its value [35]. “*They were just so different from me in terms of eating and exercising. I believe the treatment would have been more beneficial if the women in my group shared my interests*” [35]. A persistent failure to meet patient needs and expectations leads to cynicism and mistrust towards services, resulting in PTT [33].

#### Feeling unsafe in treatment

Some patients reported leaving treatment due to having had negative experiences and feeling unsafe during treatment: “*The director said, you can’t have Cheerios* […] *[the patient]* […] *took a sip of it and the director picked up the bowl and threw it across the kitchen*” [36]. Patients were admitted into care settings that were unsuitable for their needs, such as general psychiatry units or inappropriate age group mixes on wards [36]. Many felt confined by inflexible programmes, lack of freedoms, and stringent rules [39]. Feeling pressured into making changes, unhappiness with prescribed medications, poor meal choices, unhelpful punishment-and-reward systems were also referenced as [36]; and the inability to negotiate treatment plans [32]. Some felt therapy was too challenging for them, citing absence of trust in other patients within group therapy [39], feeling overwhelmed when sharing and lacked trust in the treatment process: “*I guess I never felt completely safe in the treatment setting*” [35]. Regular weighing was difficult for some patients, re-triggering obsessions with their bodies, to the point of deciding it was too much to continue [35].

### Theme 4: treatment progress

Patients prematurely left treatment due to a variety of practical and health-related obstacles that they felt limited their progress through treatment, including service provision and accessibility concerns. In some cases, patients left due to feeling they had made sufficient progress.

#### Sense of satisfactory progress

Some patients terminated treatment early due to their impression that adequate progress had been made. Some had an improvement in their condition [31, 37], believed themselves ready to continue their own weight management [38] or were happy with their progress and felt prepared to step down care [39].

#### Obstacles to treatment access

Patients reported poor service accessibility, long commutes, demanding timetables [38] and financial troubles [40]. Patients who had not completed a web-based therapy reported lack of access to technology, technical issues, long waiting times, time pressures, difficult platform design and feelings therapy had become too time-consuming [31]. Also reported as influencing decisions to terminate treatment were finding treatment elsewhere [31, 38, 40], moving away, employment-related difficulties [37], prioritisation of co-morbid conditions, pregnancy, or having acquired a different diagnosis elsewhere [37].

## Discussion

Reasons leading to PTT were found to be complex and multi-dimensional, and involved an interplay between individual, social and service-level factors. Inner conflict around enmeshment of eating disorder with identity, fractured social and clinical relationships, expectations and disappointments around treatment offered, and dissatisfaction with progress were key themes behind PTT. These themes suggest important areas for attention in both service, treatment, and research development that have the potential to improve treatment experience and recovery rates.

Theme one, ‘inner conflict’, highlighted the internal battle patients experienced when faced with a choice between the comfort and control of long-standing disordered behaviours, and entering treatment and “giving up” the disorder, something they considered intrinsic to their self-identity. The centrality of maintaining control in perpetuating eating disorders is well-reported [42, 43], and aligns with the current finding that the challenges to control and identity brought on by treatment can lead to PTT [42, 44]. Our review indicates patients found treatment threatening to their self-identity and perception of control, and in combination with other factors, such as psychiatric comorbidities, fears of weight gain and abandonment, and feeling unsupported by treatment plans and clinicians, this lead to decisions to drop out of treatment. In this context, PTT could be seen as an act of self-preservation, providing relief from these inner conflicts and a return to a long-held and dependable identity.

These findings support the notion that interventions encouraging self-worth and self-identity development should be emphasised, enabling development of deep understanding of the self, including emotional and physical needs [45]. Where patients are acutely aware of treatment challenging their identity, supporting them in reconstructing their sense of self without the eating disorder is likely to reduce PTT rates. Psychiatric co-morbidities hindered engagement and contributed to inner conflict, as eating disorders often provided a way to cope with distress. With more than 70% of eating disorder patients having co-existing mental illness [46, 47], addressing this prevalent issue early in treatment may improve PTT rates and increase the likelihood of full recovery [48, 49]

The second theme, ‘connection and communication’, illustrated the importance of developing strong patient-clinician relationships, listening to patients’ needs, supportive social networks, and the challenges difficult or intrusive family lives pose. The significance of these issues in encouraging treatment engagement has been widely recognised [20, 50–53]. Relationships established on support, empathy and respect have been shown to create positive treatment experiences and should be fostered [54]. It is noteworthy that despite the volume of literature on the importance of relationships and communication, many patients are reporting finding this lacking in their treatment. These findings again emphasise the importance of approaching treatment with a holistic understanding of the patient’s life and circumstances, and of directly enquiring about past failed treatments to determine and avoid repetition of similar patterns that previously led to PTT. This technique is often used in dialectical behaviour therapy [55], and can result in lower than average dropout rates for eating disorders [56, 57]. Supporting the patient-clinician relationship should be paramount; building a shared personal space for joint decision making, expectation setting, recognition of progress made and the nature of the therapeutic relationship explained [58, 59].

The third theme, ‘treatment service experience’, demonstrated many patients decided to leave treatment due to negative experiences and feeling unsafe within treatment, often feeling that their needs were left unmet. Treatments felt too impersonal, simultaneously restrictive and vague, provided inadequate guidance, and many felt unheard, overwhelmed and unsafe in the therapy environment. Body image, diet and weight-focussed treatments were felt to perpetuate disorders, a finding consistent with current eating disorder recovery research [60]. The importance of weight gain in some disorders cannot be denied. However, our findings imply hyper-focus on weight gain may unintentionally encourage non-engagement, and suggest services should endorse therapies addressing psychosocial and neurocognitive issues alongside weight gain. The advantages of such an approach have been recognised for improving ambivalence and treatment engagement [61, 62]. Patients felt that they would benefit from group therapies, but found that group dynamics went unmanaged by facilitators, which suggests that these therapies would benefit from facilitators skilled in this aspect. Future research should seek to investigate how specific patient-identified needs could be assimilated into existing service structures and what aspects of certain therapies (e.g. group therapy) could be improved. In already high-demand eating disorder services, treatment individuality must be balanced with cost-effectiveness and resource availability whilst maintaining quality and consistency, without compromising medical care – a difficult balance to achieve.

The fourth and final theme, ‘treatment progress’, determined that patients left treatment due to a variety of practical and health-related hurdles that they felt hindered their progress through treatment, or due to feeling that satisfactory progress had already been made. Poor service accessibility furthered PTT, compounded by long commutes and lengthy waiting times due to lack of specialist service availability [21, 63, 64]. This again highlights the importance of services and clinicians building a comprehensive picture of the patient’s life and circumstances, and of facilitating clear and open discussion from the outset around progress and discharge conditions. Difficulties accessing and using technology-based therapies, which lead to PTT, emphasised the need for well-designed, user-friendly platforms, accessible through a range of electronic devices.

Overall, the findings of this review demonstrate that psychological, behavioural, social and environmental factors are all intertwined and influence the journey through treatment, including premature termination, at both the individual and service level. This qualitative review has highlighted patient perspectives on PTT, demonstrating motivations and experiences that lead to the decision to leave treatment, and highlighting areas of focus for future research and intervention development. PTT is a key issue in eating disorder research and treatment, but as shown, also offers an invaluable opportunity to recognise the limitations of current treatments and understand who benefits and who does not, and why. Future research needs to expand on this current understanding and build knowledge of the detailed pathways determining PTT across different treatment settings.

### Strengths and limitations

This is the first review on this topic using qualitative data to explore patient experience and provides a detailed synthesis of the current evidence regarding the factors relating to PTT from eating disorder services, presenting a basis on which to develop ideas to increase treatment engagement. Further strengths include the comprehensive search strategies, broad inclusion criteria of all eating disorder diagnoses, range of treatment modalities appraised, and the use of several reviewers throughout search strategy formulation, study selection and quality assessment. Our findings demonstrate that despite heterogeneity across samples, diagnoses and treatment modalities, common experiences exist between individuals with eating disorders who do not complete treatment.

Children, men, low- and middle-income settings and ethnic minorities were underrepresented in this review, with ethnicity and socioeconomic status of participants frequently not reported in the included papers. While papers were included from multiple countries, there was a significant bias towards Caucasian participants in papers that reported ethnicity and may result in this review providing a biased view of PTT experiences, which should be taken into consideration when interpreting the findings. Future studies should include more diverse samples and address the underrepresentation of these specific groups, in order to understand cultural differences in the experience of eating disorders treatment dropout. A further limitation was the sparsity of participant quotes in some of the included articles, meaning that some supporting statements for themes in this review were based on authors’ reports rather than raw data. This, alongside the limited number of qualitative studies investigating this important issue and the limited quality of some of the included studies, suggest that a focus of future research needs to be on understanding in-depth contextual issues associated with premature termination of eating disorder treatment in each setting.

## Conclusions

In conclusion, the results of this review suggest PTT motivations are multi-faceted and wide-ranging, influenced by interactions between numerous individual, social and service-level factors. This review further highlighted the need for standardised PTT definitions, fostering a unified literature base, and continuing larger qualitative investigation into dropout experiences, with further representation across more diverse ethnic groups and gender identities. Future research and treatment services should consider these findings alongside listening to their own patients’ views and experiences to increase autonomy and individualisation, address psychosocial challenges, inform policies and improve service accessibility and delivery.

## Data Availability

Data sharing is not applicable to this article as no new data were created or analyzed in this study.
